# Broad-scale overdose education and naloxone distribution– 5-year follow-up of a regional program in Skåne County, Sweden

**DOI:** 10.1186/s12954-025-01255-3

**Published:** 2025-06-05

**Authors:** Katja Troberg, Pernilla Isendahl, Disa Dahlman, Anders Håkansson

**Affiliations:** 1 Faculty of Medicine, Department of Clinical Sciences Lund, Lund University, Lund, Sweden; 2 Malmö Addiction Center, Region Skåne, Sweden; 3 Department of Infectious Disease, University Hospital Skåne, Malmö, Sweden; 4 Center for Primary Health Care Research, Department of Clinical Sciences, Lund University/Region Skåne, Malmö, Sweden

**Keywords:** Take-home naloxone, Overdose education and naloxone distribution, People who use opioids, Harm reduction

## Abstract

**Background:**

Opioid use disorder is a chronic disorder with a high risk of overdose-related morbidity and mortality where a large proportion of these can be averted by timely administration of the antidote naloxone. For naloxone to be present when and where overdoses occur, broad-scale overdose education and naloxone distribution (OEND) must be established. A regional naloxone program was implemented in 2018, in Skåne County, Sweden. This five-year follow-up aims to describe all naloxone-related lay-person events, whether recommendations previously described in the literature were met, and to further investigate events conducted by individuals reporting overdose reversals with naloxone on three or more occasions (‘Supersavers’).

**Methods:**

Between June 2018 and June 2023, data was collected in six-month intervals from 52 participating units, containing information on trained individuals, gender, year of birth and distributed naloxone kits. Upon naloxone replenishment, patients were asked whether previous naloxone had been used for overdose reversals on someone else, or themselves, had been lost, stolen, or given to someone else. Targets for naloxone distribution and program enrolment were set to a minimum of 20 kits per annual opioid overdose death, and 100 individuals at-risk per 100,000 inhabitants, respectively.

**Results:**

Training and initial kits were provided to 2685 individuals at risk of own opioid overdose. Upon refill (*n* = 2,364), naloxone had been used for overdose reversal in 39% (*n* = 926) situations. In total, 5900 naloxone kits were distributed. Distribution target in relation to opioid overdose mortality was met annually, while the enrolment target was first met during the second year. The core group of Supersavers represent 9% (*n* = 50) of participants returning for refill while reporting 54.5% (*n* = 292) of all overdose reversals.

**Conclusions:**

Broad-scale naloxone training and distribution reaches a large proportion of individuals at risk of opioid overdose. A continuous focus and priority in supporting units with a high prevalence of individuals witnessing overdose events is of great importance as these individuals report a large proportion of overdose reversals. Likewise, it is of great importance to provide these individuals, i.e. Supersavers, with needed and sufficient support for their continued essential work intervening in overdose situations.

**Trial registration:**

Naloxone Treatment in Skåne County– Effect on Drug-related Mortality and Overdose-related Complications, NCT03570099, registered 26 June 2018.

**Supplementary Information:**

The online version contains supplementary material available at 10.1186/s12954-025-01255-3.

## Introduction

With the World Health Organization’s estimate of approximately 125,000 people dying in 2019 from opioid overdose worldwide [1] overdose mortality and morbidity persist to present a pressing public health crisis. The harms of predominantly heroin, with an increasing non-medical use of opioids such as methadone, oxycodone, buprenorphine, tramadol, fentanyl derivatives and lately also nitazenes (benzimidazole opioids), are continuously an urgent matter as these are linked to a majority (74%) of drug-related deaths in Europe [2]. International research shows a prevalence of 17–68% self-reported experience of own non-fatal overdose among people who use opioids, and that 50–96% have witnessed someone else’s overdose [3]. Naloxone hydrochloride is an opioid antagonist which has been used for decades to reverse the life-threatening effects of opioid overdose. Appropriate administration causes few negative effects and may effectively reverse opioid overdose if administered in time [4]. Besides the use of naloxone as part of emergency medical treatment in prehospital settings, implementation of regional or national large-scale overdose education and naloxone distribution (OEND) programs, offering take-home naloxone (THN) to laypersons, has increased during the last two decades [5].

On an international level, large-scale OEND programs have been successful in training potential bystanders to reverse overdose with naloxone and have been associated with reduced overdose mortality on a population level [6–8]. For naloxone to be present when and where overdoses occur, it has been estimated that OEND programs should aim to distribute 20 times the annual opiate overdose mortality rate, with a minimum of 9 times as many [9]. Research by Walley and colleagues (2013) [6] showed significant reductions in overdose deaths in communities where OEND had been implemented, with a 46% reduction of overdose deaths in communities where enrolment rates exceeded 100 per 100,000 inhabitants.

In congruence with our previous findings [10], international research found overdose reversals to be predominantly performed by individuals who themselves are at risk of overdose [11–13]. A core group of individuals, previously presented as *Supersavers* (i.e. having reported administration of naloxone on three or more occasions) [14], seems to include a rather small group of individuals who perform a large proportion of the overdose reversals. As program success generally depends on these individuals, investigation into their situation and specific needs is imperative in order to assure provision and support to maintain sustainable conditions, thus, enabling them to continue their important work.

On a national level, broad-scale provision of naloxone is imperative as the proportion of drug-related deaths in Sweden is approximately four times higher than the European average [15], of which 88% involve opioids [16]. Although guidelines on preventive training and naloxone distribution were initially published by the Swedish National Board of Health and Welfare in 2018 [17], availability and accessibility have, on a national level, generally remained insufficient and patchy with a few exceptions.

This study aims to contribute to the growing, yet scarce, body of literature on long-term follow-ups of systematic broad-scale OEND by describing all naloxone-related events during the first five years of the naloxone program in Skåne county, and in order to study whether distributed kits succeed the annual opioid overdose mortality multiplied by twenty, and the minimum of 100 enrolled per 100,000 population. Additionally, the study also aims to gather more information with respect to the core group of *Supersavers*, as they play a vital role in our regional program and its future.

## Methods

### Study design

This study is based on data from all participants who enrolled in OEND in Skåne county between June 2018 and June 2023. At the time of the study, this was the only program distributing naloxone in Skåne county. Upon completion of training and having received take-home naloxone (THN), containing two doses of intranasal naloxone (IN), year of birth, gender, facility and date of training have been registered continuously over time by key-trainers at each participating unit. For trained individuals who returned for naloxone replenishment, initial training unit was noted, and participants were asked what had happened to previous dose/-s (used on one-self, used on someone else, lost, given to other, stolen, or not known). All kits were replenished, regardless of the reason for refill of the previous kit. Participating units reported data every six months to coordinators for Skåne Naloxone program.

### Setting

Skåne Naloxone program was implemented in June 2018 and is situated in southernmost Sweden, in Skåne, a county with 1.4 million inhabitants. The program includes all opioid agonist treatment (OAT) facilities, needle and syringe programs (NSPs), in-patient addiction facilities and non-OAT outpatient facilities. The naloxone kit contains two doses of naloxone and is given to the patients directly after training, free of charge (described in detail previously in Troberg et al. (2020) [18].

NSPs in Skåne are run by the departments for infectious disease with physicians specialized in infections disease, nurses, assistant nurses, social workers, and midwives offering comprehensive free of charge healthcare including tests for blood borne viruses, vaccination for hepatitis A and B, and hepatitis C treatment besides providing visitors with clean needles and syringes.

Only facilities specialized in psychiatry may provide OAT. On a national level, a survey referred to by The National Board of Health and Welfare showed that approximately 65 per 100,000 inhabitants were enrolled in OAT in 2022 [19], with low availability in rural areas. All regions are self-governing. However, in Skåne county, the situation is unique compared to the rest of Sweden, as OATs are run by both public and privately owned healthcare providers, through a “choice of healthcare” system, rendering a much higher availability at 158/100,000 (data collected July 2023 from all OAT units in Skåne by authors KT and PI). Swedish healthcare and medication are heavily subsidised by governmental fundings.

Naloxone is provided free of charge at all facilities included in the program. During the study period, naloxone could be prescribed by any physician. As of November 1^st,^ 2018, naloxone could also be prescribed by registered nurses. As with the majority of all prescribed medication, naloxone included in the high-cost protection scheme, a subsidy that sets an upper limit of individual costs for medication and medical care [20].

### Study participants

All individuals (*n* = 2685) took part of the theoretical and practical overdose prevention training and were provided with a naloxone-kit from one of the OEND facilities (*n* = 52) in Skåne county. The study included the following facilities in the region: NSPs (*n* = 4), OATs (*n* = 30), emergency ward (*n* = 1), in-patient addiction units, non-OAT out-patient addiction units and psychiatric facilities (*n* = 17). Three of the OAT facilities closed during the study period but provided data for the study until closure. Patients were referred to other OAT facilities in the region. Information on whether they had been enrolled in OEND was obtained by the OAT facility referred to, to ensure that participants were only registered during the initial training. While repeat training was available for all participants throughout the 5-year period, these were not registered for the purpose of this study. An additional map shows the location of units in more detail [see Additional File 1, Supplementary Figure A].

Throughout this study we refer to participants having reported naloxone use for overdose reversals on three occasions or more as “Supersavers”, a definition previously applied in similar research settings in our neighboring country Norway [14].

While preventive training has been provided to the broader community, this study only presents data concerning individuals at risk of own overdose who received naloxone through the regional program, as to this date, naloxone has not been available to others.

### Procedures and data management

Data regarding all individuals participating in OEND were collected on a regular basis (every six months) from key instructors at all participating units. Program coordinators (KT and PI) provided a template (pen-and-paper or digital) for each unit to report all events concerning training, inclusion and reason for naloxone replenishment Program coordinators then transferred all new events to an Excel file unique to every unit. This was returned to the key instructors for control whereafter reported data accuracy was confirmed by telephone. Data collected from all the units during the five-year period was compiled into an Excel file and transferred to SPSS for analysis. Variables included unit providing initial training and naloxone, type of unit, city/town, date of inclusion, year of birth, gender and numbers of kits issued for replenishment, and what had happened to the previous kit (lost/stolen/given to someone else/used on someone else/ used on oneself/not known). Additional variables were constructed: Returned for refill, naloxone used (used on someone else or oneself), Supersaver (having used naloxone for overdose reversal on three or more occasion), non-Supersaver (having used naloxone 0–2 times).

Reports of refills due to previous naloxone having expired were excluded from the total number of refills as a large batch with short expiry date had been distributed and consequently had to be replaced within 6 months. Although most expired doses were replaced, some were simply given additional doses. To certify that the numbers of kits out in the public are not exaggerated, these were excluded from the analysis.

### Outcome measures

The following measures were examined: Number of trained individuals, reasons for naloxone replenishment (including reports of overdose reversals), and Supersaver contribution. The overall OEND distribution rate was described using the target previously suggested by Bird et al. (2015) [9] as 20 times the number of annual opiate-related fatalities whereas the enrolment target was set to a minimum of a 100 per 100,000 population, as previously suggested by Walley et al. (2013) [6].

The analysis of distribution in relation to rates of opioid related deaths in Skåne County, were defined as annual deaths diagnosed according to the International Classification of Diseases, 10th revision (ICD-10) as either F11.0 (acute opioid intoxication), X42 (accidental narcotics intoxication) and Y12 (narcotics intoxication with unknown intent), aged 15 and older, in the national causes of death register. To estimate targets for distribution in relation to annual death, the mean number of annual deaths for the five-year period prior to the specific year was applied (i.e. for distribution year 2018–2019, the mean number of opioid overdose deaths between 2013 and 2017 was used, and so forward).

### Statistical analysis

P-values < 0.05 with 95% CI was found to be statistically significant. The collected data was analyzed using SPSS Statistics Version 29.0 [21] and analyzed through descriptive statistics, chi-2-test and Students’ t-test.

## Results

### Participants characteristics

Preventive overdose training and an initial kit containing two doses of naloxone were provided to 2,685 participants. The majority of participants (66%, *n* = 1,782) were recruited from OAT facilities, while patients recruited at NSPs, or in-patient facilities, represented 17% respectively [Table 1].

**Table 1:** Baseline characteristics of individuals receiving initial overdose education and take-home Naloxone (*n* = 2685)

	*N* (%)
**Unit providing training and initial kit**	
Needle and syringe program	459 (17.1)
Opioid agonist treatment	1782 (66.4)
In-patient	444 (16.5)
**Gender** ^a^	
Male	1858 (70.6)
Female	773 (29.4)
**Age** ^b^	
Mean age (years/SD)	39.5 (11.5)
Range	56 (18–74)
Median age (IQR; Q1-Q3))	38.0 (17; 30–47)
**Year of inclusion**	
2018	519 (19.7)
2019	718 (27.2)
2020	435 (16.5)
2021	435 (16.5)
2022	374 (14.2)
2023	160 (6.1)

*Patients in outpatient addiction treatment facilities included in this group (non-OAT) *n* = 54

^a^ missing *n* = 23, ^b^ missing *n* = 77

The cohort represented individuals between the ages of 18 and 74 years, with a median age of 38 years and interquartile range (IQR) of 17 (Q1 = 30 and Q3 = 47). 71% were male. [Table 1]. While median age among patients recruited from OAT (39 years, IQR 18) was slightly higher than that of participants recruited from NSP (38 years, IQR 17), a younger cohort appeared to have been reached through in-patient facilities as median age was found to 32 years (IQR 16). For more details, please see the additional table [please see Additional file 2, Supplementary Table A].

Although implementation started in June 2018 implying a start-up period in the middle of the summer holidays, 20% of all participants were included during the first six months. During the last six months, between January and June 2023, only 6% were included, as units found it increasingly harder to recruit new participants.

### Reason for Naloxone refill, use and type of unit

During the five-year period, 2364 refills were reported of which a total number of 926 overdose reversals were performed. The majority (93%) had been used for overdose reversal on someone other than the person to whom naloxone had been prescribed. Although most patients had been provided training and THN at an OAT facility, significantly more reversals on others were reported by participants trained at, and returning to, NSP for refill (*p* < 0.01), compared to reversals by those trained at, and returning to, OATs. No significant difference was found between the two groups regarding reversals on the person that had reported the incident. Significant differences between groups were found regarding reports on naloxone lost or given to other (*p* < 0.01 and *p* = 0.04, respectively) [Table 2]. Mean number of refills per trained individual were 2.8 at NSPs while 0.6 at OATs, while the average number of overdose reversals per trained individual were 1.4 among participants trained at NSPs, in contrast to 0.2 for those trained at OAT.

**Table 2:** Naloxone replenishment, use of previous Naloxone and type of facility providing refill (*N* = 2364)

	All refills *N* (%)	NSP refills *N* (%)	OAT refills *N* (%)	95% CI Mean diff (Lower/Upper)	t-test (2-sided) *p*-value
Refills	2364	1294	983	1.416 (1.217—1.614)	< 0.01*
Overdose (OD) reversal	926 (39.2)	621 (48.0)	281 (28.6)	1.008 (0.706—1.311)	< 0.01*
Reversal of OD other	857 (36.3)	571 (44.0)	262 (26.7)	0.967 (0.660—1.275)	< 0.01*
Reversal OD on self	69 (2.9)	50 (3.9)	19 (1.9)	0.164 (-0.220—0.548)	0.396
Lost	741 (31.3)	431 (33.3)	289 (29.4)	0.579 (0.374—0.783)	< 0.01*
Given to other	328 (13.9)	143 (11.1)	179 (18.2)	0.270 (0.014—0.525)	0.04*
Other (incl. stolen)	77 (3.3)	58 (4.5)	18 (1.8)	0.101 (-0.115—0.317)	0.353
Not known	292 (12.4)	41 (3.2)	216 (22.0)	0.020 (-0.107—0.147)	0.760

Expired doses were excluded (*n* = 851 in relation to all refills, of which *n* = 92 and *n* = 749 at NSPs and OATs respectively)

In-patient refills (*n* = 87) have been excluded from this analysis due to their low numbers

*p = < 0.05

### Supersavers

A first analysis of the material identified 74 cases where individuals had used naloxone on three or more occasions for overdose reversal, of which 50 could be traced throughout the entire period, eliminating cases for those who may have reported three or more overdose reversals at multiple units.

Although the proportion of women among Supersavers (38%) was higher than among non-Supersavers (31%), the difference was not significant (*p* = 0.33), nor were there any significant differences in age between the two groups (*p* = 0.87). Access to training and refill through NSPs were a significant predictor of being a Supersaver (*p* < 0.01).

Compared to non-Supersavers, Supersavers more often reported previously having administered naloxone on someone else (*p* < 0.01), that naloxone had been administered on themselves by someone else (*p* < 0.01), and having lost their previous naloxone kit (*p* < 0.01). Reports of previous naloxone as given to someone else, stolen/other, or not known, did not differ significantly between groups [Table 3].

Among male Supersavers, the mean number of overdose reversals was 5.1, while women had reported an average of 6.2.

**Table 3:** Baseline characteristics and reason for refill, for participants returning, comparing non-Supersavers and supersavers

	Return for refill (all) (*n* = 560)	Non-Supersavers (naloxone use 0–2 times) (*n* = 510)	Supersavers (Naloxone used ≥ 3 times) (*n* = 50)	*p*-value
Male n (%)	377 (68.1) ^a^	346 (68.7) ^a^	31 (62.0)	0.336
Female n (%)	177 (31.9) ^a^	158 (31.3) ^a^	19 (38.0)	
Mean age (SD)	39.8 (10.5) ^a^	39.8 (10.4) ^b^	39.6 (11.4)	0.888
Median age (IQR; Q1–Q3)	38 (16; 31–47) ^a^	38 (16; 31–47) ^b^	36.5 (18; 31–49)	
Initial training NSP n (%)	230 (41.1)	186 (36.5)	44 (88.0)	< 0.001*
Initial training OAT n (%)	319 (57.0)	313 (61.4)	6 (12.0)	
Initial training In-patient n (%)	11 (2.0)	11 (2.2)	0	
Refills (n)	1208	820	388	< 0.001*
Mean number of refills (range)	2.16 (21; 1–22)	1.61 (9; 1–10)	7.76 (19; 3–22)	
Naloxone used for overdose reversal n (%)	536 (44.4)	244 (29.8)	292 (75.3)	< 0.001*
Naloxone use on others n (%)	490 (40.6)	215 (26.2)	275 (70.9)	< 0.001*
Mean OD reversals on others (range)	0.88 (21;1–22)	0.42	5.50 (19;3–22)	
Naloxone used on one-self n (%)	46 (3.8)	29 (3.5)	17 (4.4)	0.007*
Mean OD reversals on one-self (range)	0.08 (3;1–4)	0.06 (1;1–2)	0.34 (3;1–4)	
Lost n (%)	339 (28.1)	276 (33.4)	63 (16.2)	< 0.001*
Given n (%)	169 (14.0)	146 (17.8)	23 (5.9)	0.064
Stolen/other n (%)	39 (3.2)	31 (3.9)	8 (2.1)	0.334
Not known n (%)	125 (10.4)	123 (15.0)	2 (0.0)	0.848

Participants returning for refill at a different unit than the unit which had provided initial training were excluded (loss to follow-up)

Refills due to previous naloxone having expired have been excluded (*n* = 851)

*p = < 0.05 Pearson Chi-2-test for all analyzes except for age, for which Student’s T-test was applied

^a^ Missing *n* = 6, valid percent

### Annual OEND enrolment and THN distribution in relation to recommendations

Although annual distribution exceeded the recommended target of 20 times the annual opioid overdose deaths [9], the cumulative enrolment target on training and distribution did not exceed 100 per 100,000 inhabitants [6] until the second year [Table 4].

**Table 4:** Annual overdose education enrolment and Naloxone distribution in relation to previously suggested targets ^a^^, c^

	1st year June 2018–June 2019	2nd year June 2019–June 2020	3rd year June 2020– June 2021	4th year June 2021– June 2022	5th year June 2022– June 2023
**Distribution**					
Annual distribution, Skåne county	1045	1060	730	961	1253
Suggested annual distribution in relation to overdose deaths ^a, b^	380–844	371–824	353–784	315–700	297–652
Cumulative distribution	1045	2105	2835	3796	5049
Annual distribution in per 100,000 inhabitants ^d^	76	77	52	68	88
Cumulative distribution per 100,000 inhabitants ^d^	76	152	203	270	356
**Enrolment**					
Annual enrolment, Skåne county	892	663	313	376	441
Annual enrolment per 100,000 inhabitants in Skåne county ^c, d^	65	48	22	27	31
Cumulative enrolment	892	1555	1868	2244	2685
Cumulative enrolment rates per 100,000 inhabitants in Skåne county ^c, d^	65	113	135	162	193

Refills due to previous naloxone expired have been excluded, *n* = 851

^a^ Targeting to distribute 20 times the number of annual opiate-related fatalities, and a minimum of 9 times annual fatalities, rates suggested by Bird et al. (2015) [9] as crucial for naloxone to be present when overdoses occur

^b^ The average opioid related deaths for the 5-year period prior to measuring year among individuals in Skåne County, aged 15 and older at the time of death, with X42 or Y12 diagnoses were 42.2 individuals in 2013–2017, respectively 41.2 and 39.2 during 2014–2018 and 2015–2019, and 35.0, respectively 32.6 during 2016–2020 and 2017–2021 (National causes of death register)

^c^ Walley et al. (2013) [6] Targeting enrolment of 100, or more, individuals in OEND per 100 000 population

^d^ Population in Skåne https://www.statistikdatabasen.scb.se/ 2018–2019: 1,369,996, 2019–2020: 1,383,582, 2020–2021:1395881, 2021–2022: 1,408,375, 2022–2023: 1,418,053

For detailed information regarding training, distributed kits and reports of naloxone use for overdose reversals, including data from each 6-month interval and accumulated data throughout the full 5-year study period, please see supplementary material [Additional file 3 and 4, Supplementary Figure B and C].

## Discussion

Since program start in June 2018, naloxone distribution has increased due to effective implementation into an already existing infrastructure of needle and syringe programs and addiction facilities [18]. The annual naloxone distribution exceeded the target of 20 times annual opioid overdose deaths recommended by Bird et al., [9], although the annual target of 100 enrolments or distributed kits per 100,000 inhabitants was not met, whereas both the cumulative enrolment and distribution successfully exceeded 100 per 100,000 inhabitants [6] during the second year. Although these numbers may be somewhat disappointing, our previous assessment of the program’s effect on mortality during the intervention period (2019–2021), compared with the historic control period (2013–2017), did present a significant decrease [8]. Although the cost of naloxone and additional material was covered by the region, there was no addition to the workforce when it came to training and distribution of naloxone for patients. For efficiency, individuals with own risk of overdose were prioritized, as recommended by previous research [22].

Upon naloxone replenishment (expired doses excluded), there were 926 (39%) reports of naloxone being used for overdose reversals. Had the expired naloxone been included, the percentage would have dropped to 29%, a somewhat lower percentage than that of 60% reported in our previous 30-month follow-up. In part, the difference could be explained by the expansion of the program, initially targeting individuals with a higher risk of overdose involvement, where the expansion may have subsequently reached individuals with lower degree of risk behavior. With different settings providing different challenges, such as availability and potency of illicit drugs, legal barriers, stigma, economy, access and availability of health care services, comparisons of naloxone use in relation to distribution between programs, may be difficult. The systematic review by McDonald and Strang [23] showed a broad variation of used naloxone in relation to total distribution (4–67%), where studies with more than 1500 distributed kits showed a variation between 9 and 28%, compared to the 16% found in Skåne county, if expired doses were to be included.

Fear of negative consequences, such as losing take-home medications privileges among participants reporting overdoses at OAT, may result in an underreporting of naloxone used in OD reversals. Fear of social welfare being contacted if suspected of drug use while caring for minors may also be of general concern among participants, and this has been described as a barrier especially for women when it comes to seeking treatment and/or harm reduction services [24, 25].

In spite of a steady increase of OAT patients in Skåne during the study period, from 1579 to 2226, 79% had been enrolled in OEND in June 2023. Although the majority of OEND participants had received training through OATs, overdose reversals were more frequently reported among participants returning to NSPs for refill. This is not surprising as individuals who actively use drugs are more likely to witness more overdoses [6, 10, 11, 14, 22]. Compared to the 30-month follow-up, an increase in overdose reversals were reported at NSPs (67.1% vs. 53.7%). An increase in participants trained at in-patient facilities was seen, from 11 to 17%, while the number of patients trained at NSPs changed modestly from 15 to 17% at NSPs, and the number of patients trained at OAT was reduced from 74 to 66%. This implies a continuously high engagement by NSP visitors. These numbers imply the importance of a constant need for NSPs to be prioritized as main hubs for overdose prevention training and naloxone distribution [10]. Although there is a reduced risk of overdose among OAT patients [26], NSPs and OATs serve as a continuum rather than two separate entities.

As previously described by Bennett et al. [27], people who use drugs and their peers often turn to “nodes” or “hubs” of individuals recognized to possess increased experience and knowledge and may therefore serve as an effective link from mainstream healthcare to hard-to-reach individuals [27]. This implies that through these hubs, training and naloxone distribution could also reach at-risk individuals efficiently. More recent data from Norway found Supersavers to be younger, more commonly using opioids actively and having witnessed more overdoses at baseline than non-Supersavers. Neither our nor the Norwegian Supersavers showed any significant differences in gender [14]. No significant age difference was found between Supersavers and non-Supersavers, nor was the Norwegian findings concerning opioid use and having witnessed an increased number of overdoses [14] found among our participants. However, having received initial training and naloxone kit through NSPs was significantly more common among Supersavers (*p* < 0.01), and this may serve as a proxy for being more actively involved in substance use.

The lack of significant results may at least partly be explained by a large percentage being lost to follow up (33%). Supersavers represented less than 2% of all trained individuals, and less than 9% of those returning for refill however reported more than half (54%) of all reversals registered at initial training unit. This calls for a need to secure adequate and requested support for these individuals´ energy and motivation to continue their important efforts to keep peers, family members and acquaintances alive.

Skåne Naloxone program has shown that effective multi-site implementation of THN is possible, even in Sweden, a country traditionally identified as a high-threshold nation with respect to harm reduction interventions. This model can easily be implemented in other settings and the program continues to expand. Between the 30-month and the 5-year follow-up, the program was implemented at an additional seven units. Allowing for future rapid upscaling, an educational film on overdose prevention and practical training has been launched. In an effort to further broaden access to naloxone The Swedish Medical Products Agency announced for Naloxone to be made available at pharmacies over the counter during 2024 [28].

There are several limitations to this study. The study is based on self-reported data and may be subject to recall and social desirability bias. Fear of stigma, or fear of losing benefits of take-home OAT-medication, may also lead to individuals preferring to report previous doses as lost, not known or given to someone else rather than being used in reversals on oneself. Data was only available for those returning for refill, and therefore, overdose events may have been missed as there was no other active follow-up.

As data initially was collected primarily for clinical follow-up purposes, there was no identification number which could be traced. This meant that a rather high proportion of events had to be counted as “lost to follow-up” as only those returning to the unit where they had received their initial training could be followed. Also, the definition of a supersaver was based on reports from the initial training unit whereby data of additional refills made at other units was lost on an individual level. The actual number of Supersavers may therefore be higher than reported here.

Although Sweden was not at any time during the Covid 19-pandemic subject to formal lock-down procedures, however, it is likely that the increased strain on healthcare in general also had a negative effect on program enrolment and naloxone distribution.

Despite these limitations, through this data we were able to follow a large proportion of the events reported over the first five years of the regional program with 2685 participants having received take-home naloxone, theoretical and practical overdose prevention training.

## Conclusions

Large scale implementation of overdose education and naloxone distribution using the existing infrastructure of low-threshold facilities effectively recruits people in sufficiently high numbers to meet the proposed target values from previous studies and effectively reaches individuals at risk of witnessing overdoses. OAT facilities are continuously a major provider of training to at-risk individuals, though reversals primarily are reported by patient returning to NSP for refills, which highlights the importance of a continuous need for NSPs to be prioritized when it comes to overdose prevention training and naloxone distribution. As a small proportion of enrolees represent a large proportion of reversals, their actions are of great importance to their community and to their peers.

## Electronic supplementary material

Below is the link to the electronic supplementary material.

Supplementary Material 1: Additional file 1- Supplementary Figure A. Units included in Skåne Naloxone program, June 2018– June 2023.

Supplementary Material 2: Additional file 2 - Supplementary Table A. Baseline characteristics of individuals receiving training and naloxone.

Supplementary Material 3: Additional file 3 - Supplementary Figure B. OEND Training, distributed kits and reports of previous naloxone used for overdose reversals, June 2018 - June 2023, 6-month intervals.

Supplementary Material 4: Additional file 4 - Supplementary Figure C. Accumulated OEND Training, distributed kits and reports of previous naloxone used for overdose reversals, June 2018 - June 2023, 6-month intervals.

## Data Availability

The SPSS data used to support the findings of this study are restricted by the Regional Ethics Board, Lund, Sweden, to protect people’s privacy. The data are not publicly available due to the sensitivity of substance use and due to participant privacy concerns. Though, data is available from the corresponding author on request, for researchers who meet the criteria for access to confidential data.

## Abbreviations

IN: Intranasal Naloxone
NSP: Needle and Syringe Program
OAT: Opioid Agonist Treatment
OEND: Overdose Education and Naloxone Distribution
THN: Take-Home Naloxone
