# Supplementary material for: Broad-scale overdose education and naloxone distribution– 5-year follow-up of a regional program in Skåne County, Sweden
Source: Harm Reduct J. 2025 Jun 5;22:97. doi: 10.1186/s12954-025-01255-3 (PMC12139078; doi:10.1186/s12954-025-01255-3)
Supplement: Supplementary file 1 — Supplementary Material 1: Additional file 1- Supplementary Figure A. Units included in Skåne Naloxone program, June 2018– June 2023. [file 12954_2025_1255_MOESM1_ESM.doc]

**Additional file 2.**

| **Supplementary Table A. Baseline characteristics of individuals receiving training and take-home naloxone** | | | | | |
| --- | --- | --- | --- | --- | --- |
|  | | All participants | NSP | OAT* | In-patient |
| Number of trained individuals n (%) | | 2685 | 459 (17.1) | 1782 (66.4) | 444 (16.54) |
| Median age (IQR; Q1-Q3) | | 38 (17; 30-47) | 38 (17; 31-48) | 39 (18; 31-49) | 32 (16; 26-42) |
| Mean age (Std. dev) | | 39.5 (11.5)  ^Missing n=77^ | 39.5 (11.2)  ^Missing n=2^ | 40.6 (11.4)  ^Missing n=50^ | 34.7 (11.2)  ^Missing =25^ |
| Men n (%) | | 1858 (70.6)  ^Missing n=23^ | 310 (67.5) | 1233 (70.4)  ^Missing n=31^ | 315 (74.8)  ^Missing n=23^ |
|  | Median age (IQR; Q1-Q3)  (men) | 38 (17; 31-48)  ^Missing n=42^ | 38 (18; 32-50)  ^Missing n=2^ | 40 (17; 32-49)  ^Missing n=37^ | 32 (16; 26-42)  ^Missing n=3^ |
| Women n (%) | | 773 (29.4)  ^Missing n=54^ | 149 (32.5) | 518 (29.6)  ^Missing n=31^ | 106 (25.2)  ^Missing n=23^ |
|  | Median age (IQR; Q1-Q3)  (women) | 37 (16; 30-46)  ^Missing n=13^ | 36 (16; 28-43) | 38 (16; 31-47)  ^Missing n=12^ | 32 (17; 25-42)  ^Missing n=1^ |
| *Patients in outpatient addiction treatment units included (non-OAT) n=21 | | | | | |
