# Supplementary material for: Broad-scale overdose education and naloxone distribution– 5-year follow-up of a regional program in Skåne County, Sweden
Source: Harm Reduct J. 2025 Jun 5;22:97. doi: 10.1186/s12954-025-01255-3 (PMC12139078; doi:10.1186/s12954-025-01255-3)
Supplement: Supplementary file 2 — Supplementary Material 2: Additional file 2 - Supplementary Table A. Baseline characteristics of individuals receiving training and naloxone. [file 12954_2025_1255_MOESM2_ESM.doc]

Additional File 3. Supplementary Figure B.


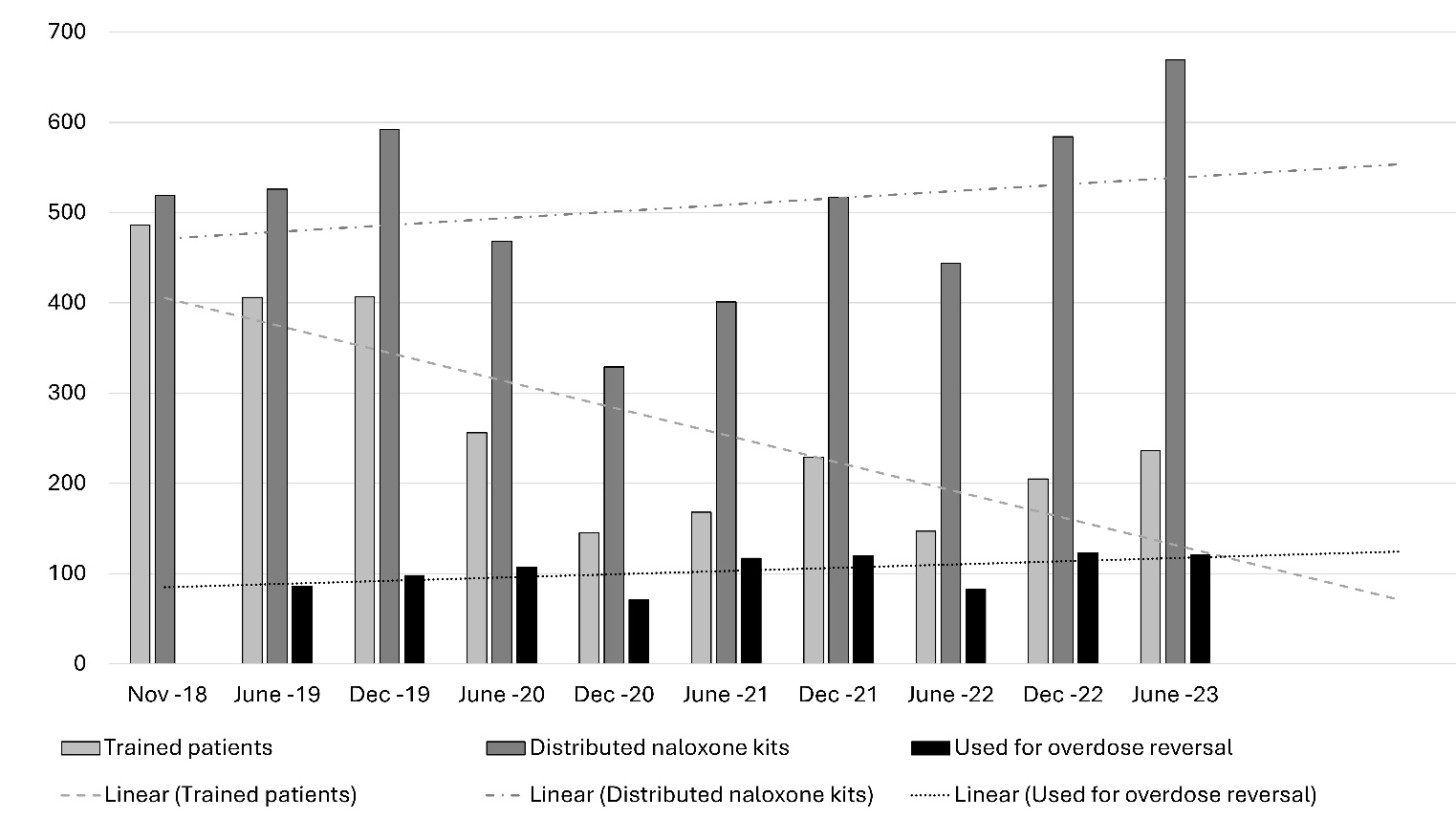


**Figure B. OEND Training, distributed kits and reports of previous naloxone used for overdose reversals, June 2018 - June 2023, 6-month intervals (refills due to expiry date have been excluded).**

After exclusion of all refills due previous naloxone having expired (n=851), a total number of 5049 kits each containing two doses of IN naloxone had been distributed to 2685 trained individuals, between June 2018 – June 2023. Figure B, above, illustrates an overview of patients trained, naloxone distributed, and reports of naloxone used for overdose reversal during 6-month intervals.
