# Supplementary material for: Broad-scale overdose education and naloxone distribution– 5-year follow-up of a regional program in Skåne County, Sweden
Source: Harm Reduct J. 2025 Jun 5;22:97. doi: 10.1186/s12954-025-01255-3 (PMC12139078; doi:10.1186/s12954-025-01255-3)
Supplement: Supplementary file 3 — Supplementary Material 3: Additional file 3 - Supplementary Figure B. OEND Training, distributed kits and reports of previous naloxone used for overdose reversals, June 2018 - June 2023, 6-month intervals. [file 12954_2025_1255_MOESM3_ESM.doc]

**Additional file 1. Supplementary Figure A. Units included in Skåne Naloxone program, June 2018 – June 2023.**

All individuals (n=2685) took part of the theoretical and practical overdose prevention training and were provided with a naloxone-kit from one of the OEND units (n=52) in Skåne county. The study included the following units in the region: NSPs (n=4), OATs (n=30), emergency ward (n=1), in-patient addiction units, non-OAT out-patient addiction units and psychiatric facilities (n=17) [Figure 1], of which three OAT facilities closed during the study period.


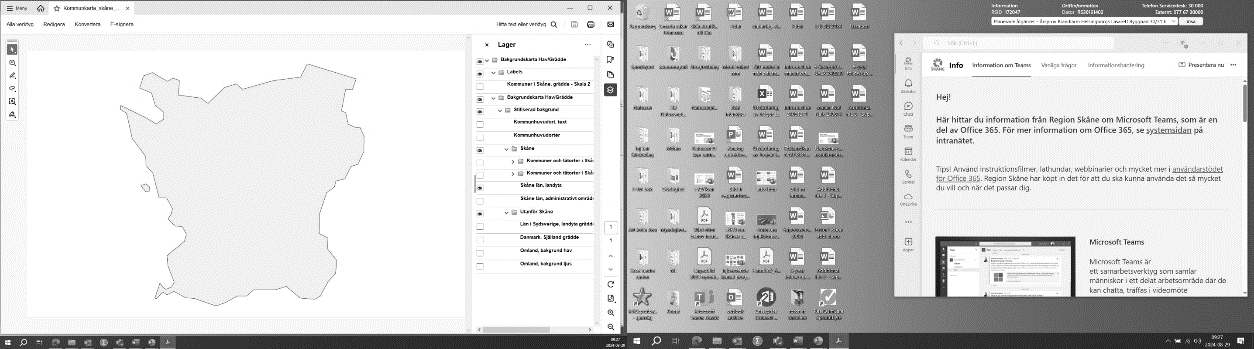


NSP

OAT (of which 3 units have closed)

Addiction and psychiatric facilities (non-OAT)

Emergency care unit

Figure 1. Units included in Skåne Naloxone program, June 2018 – June 2023.
