# Supplementary material for: Broad-scale overdose education and naloxone distribution– 5-year follow-up of a regional program in Skåne County, Sweden
Source: Harm Reduct J. 2025 Jun 5;22:97. doi: 10.1186/s12954-025-01255-3 (PMC12139078; doi:10.1186/s12954-025-01255-3)
Supplement: Supplementary file 4 — Supplementary Material 4: Additional file 4 - Supplementary Figure C. Accumulated OEND Training, distributed kits and reports of previous naloxone used for overdose reversals, June 2018 - June 2023, 6-month intervals. [file 12954_2025_1255_MOESM4_ESM.doc]

Additional File 4. Supplementary Figure C.

**
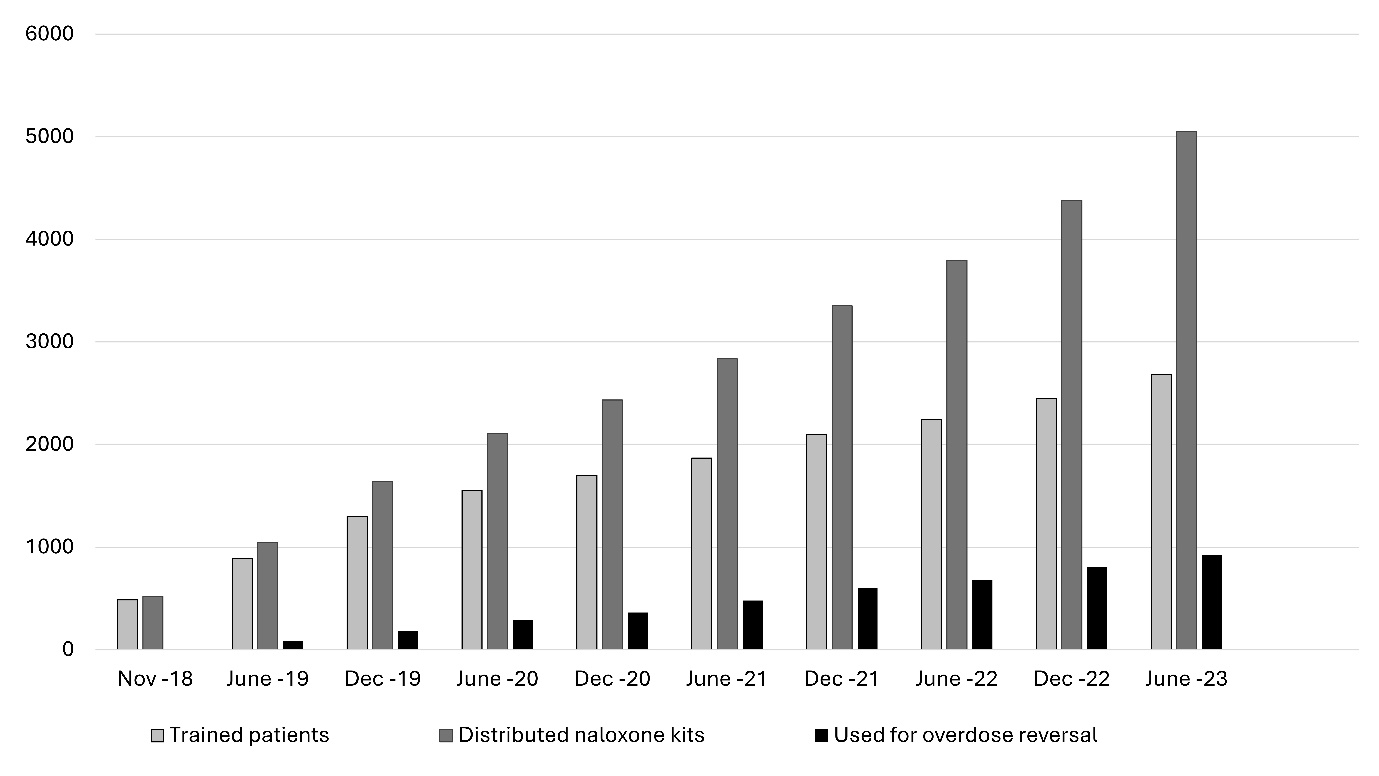
**

**Figure C. Accumulated OEND Training, distributed kits and reports of previous naloxone used for overdose reversals, June 2018 - June 2023, 6-month intervals (refills due to expiry date have been excluded).**
